# Supplementary material for: MetaRibo-Seq measures translation in microbiomes
Source: Nat Commun. 2020 Jun 29;11:3268. doi: 10.1038/s41467-020-17081-z (PMC7324362; doi:10.1038/s41467-020-17081-z)
Supplement: Supplementary file 10 — Supplementary Data 7 [file 41467_2020_17081_MOESM10_ESM.zip › File2/Confidence_High_Taxonomy/287105_out.krona.html]

Javascript must be enabled to view this page.

members
magnitude
magnitudeUnassigned
count
unassigned
taxon
rank

287105\_out


SRS1054712\_contig\_number\_contig-100\_617.618SRS1054716\_contig\_number\_contig-100\_11579.11580SRS1055054\_contig\_number\_2675
3
6

superkingdom
3
2

1
phylum
1224

1236
class
1

91347
order
1

1
family
543

561
genus
1


SRS1054691\_contig\_number\_contig-100\_619.72010
species
1
562

1239
phylum
2

class
2
91061

186826
order
2

81852
2
family

1350
2
genus

SRS142531\_contig\_number\_contig-100\_29923.29923SRS149671\_contig\_number\_contig-100\_32779.64668
